# Adapting community-based sexual and reproductive health services for adolescents and young people aged 15-24 years in response to COVID-19 in Lusaka, Zambia: the implications on the uptake of HIV testing services

**DOI:** 10.1186/s12913-022-07878-7

**Published:** 2022-04-14

**Authors:** Mwelwa Muleba Phiri, Bernadette Hensen, Ab Schaap, Lucheka Sigande, Melvin Simuyaba, Musonda Simwinga, Sian Floyd, Sarah Fidler, Richard Hayes, Helen Ayles

**Affiliations:** 1 Zambart, UNZA Ridgeway Campus, off Nationalist Road, Lusaka, Zambia; 2 Clinical Research Department, London School of Hygiene and Tropical Medicine, London, UK; 3 Department of Infectious Disease Epidemiology, London School of Hygiene and Tropical Medicine, London, UK; 4 Imperial College and Imperial College NIHR BRC, London, UK

**Keywords:** Adolescents and young people, COVID-19, HIV testing, Sexual and reproductive health, Zambia, Community-based

## Abstract

**Background:**

Across Sub-Saharan Africa, adolescents and young people (AYP) aged 15-24 have limited access to sexual and reproductive health (SRH) services, including HIV testing services (HTS). In response, the Yathu Yathu study was implemented in two high-density communities in Lusaka, Zambia. Yathu Yathu provides comprehensive, community-based, peer-led SRH services, including differentiated HTS (finger-prick and HIV self-testing) and comprehensive sexuality education (CSE). We describe adaptations to the Yathu Yathu intervention in response to the COVID-19 epidemic, and implications on uptake of HTS among AYP.

**Methods:**

Yathu Yathu provides SRH services through community-based peer-led spaces. AYP in study communities were offered prevention points cards (PPC), which incentivizes and tracks service use. Social media (WhatsApp©/Facebook©) is used to engage and inform AYP about SRH. Due to COVID-19, hubs closed from April-June 2020. We describe adaptations in response to COVID-19 and, using routinely collected PPC data, describe uptake of HTS before (September 2019-March 2020) and after (July-December 2020) adaptations in response to COVID-19. We describe reach of the Yathu Yathu Facebook page and use qualitative data to describe AYP experiences of SRH service access.

**Results:**

During hub closures, CSE was delivered via video on social media, resulting in an increase in Facebook page followers from 539(April) to 891(June). WhatsApp groups evolved as a platform to deliver CSE and COVID-19 information, with higher participation among young people aged 20-24. Key service delivery adaptations included: reducing the number of participants in hubs, mandatory handwashing before entry, use of personal protective equipment by staff and provision of facemasks to AYP. HTS were provided as normal. Adaptations led to fewer AYP attending hubs. Uptake of HTS among AYP visiting hubs for the first time after COVID-19-related closures was higher (73.2%) compared to uptake before adaptations (65.9%; adjOR=1.24 95%CI 0.99, 1.56, *p*=0.06). Despite disappointments with some aspects of service delivery, AYP expressed happiness that hubs had reopened.

**Conclusions:**

Social media can be a useful additional platform to reach AYP with HIV prevention information during COVID-19. With proper infection control in place, HTS can safely be provided to, accessed and accepted by AYP in community-based settings during COVID-19.

**Trial Registration:**

National Clinical Trials NCT04060420,19^th^ August 2019. Current Controlled Trials ISRCTN75609016, 14^th^ September 2021, retrospectively registered.

## Background

Sub-Saharan Africa, particularly Eastern and Southern Africa, is disproportionately affected by HIV. Of the estimated 5000 daily new infections globally in 2018, approximately 61% were in Sub-Saharan Africa. A third of these infections were among adolescents and young people (AYP) aged 15-24. In Eastern and Southern Africa, 1 in 4 new infections were among adolescent girls and young women (AGYW) aged 15-24, who account for only 10% of the population [1]. In addition to a high risk of HIV infection, AYP, especially AGYM, are at high risk of other sexually transmitted infections (STIs); though at lower risk compared to young women, young men report higher risk sex and are less engaged with health services [2–5]. This data points to a need for information and education on STIs/HIV, such as through provision of comprehensive sexuality education (CSE), and access to diagnostic services, including HIV testing services (HTS).

Data on access to essential sexual and reproductive health (SRH) services, including HTS, shows AYP are underserved. In Zambia, the age group 15-19 have the lowest coverage of HIV testing and correct knowledge of HIV: 59% of girls and 46% of boys aged 15-19 reported ever testing compared to 91% of women and 77% of men aged 20-24yrs [6]. Access is hindered by numerous individual-level and structural-level factors, including negative staff attitudes, and lack of youth-friendly services [7, 8]. In Zambia, a community-based door-to-door universal HIV testing-and-treatment community-randomized study (HPTN071/PopART) found gaps in reaching AYP, particularly young men aged <25 [9]. To address these gaps, the P-ART-Y study was nested within PopART to reach adolescents. P-ART-Y found that knowledge of HIV status in intervention communities was >80% compared to ˜30% in control communities; while better than regional estimates, this still leaves many AYP unreached by HTS [10–12]. In response to these unmet needs, the Yathu Yathu (“For us, by us”) intervention was co-designed with AYP to provide community-based, peer-led SRH services for AYP aged 15-24 in two urban communities in Lusaka, Zambia [13].

In March, 2020, the World Health Organization declared the SARS-CoV-2(COVID-19) outbreak a “public health emergency of international concern” [14]. The first case in Zambia was reported on 16^th^ March, 2020 resulting in closure of schools, mandatory mask-wearing and restrictions of public gatherings, among other measures [15]. Early estimates of the impact of COVID-19 predicted that access to SRH services would be negatively affected, this conclusion was drawn from lessons learnt during outbreaks of other diseases, such as Ebola [16–18] and cholera. Using data collected in the Yathu Yathu study, we describe adaptations to service delivery in response to the COVID-19 epidemic, implications on AYP’s access to Yathu Yathu and uptake of HTS, and AYP’s perceptions and experiences of accessing SRH services adapted in response to COVID-19.

## Methods

### Study location and population

The impact of Yathu Yathu (‘for us, by us’) on knowledge of HIV status is being evaluated using a cluster randomized trial (CRT), details of the CRT are described elsewhere [19]. Briefly, the CRT is being conducted in two densely populated urban communities in Lusaka, which were split into 10 zones each, with each zone having a population of ˜2350 AYP. These 20 zones were randomly allocated, with an allocation ratio of 1:1, to intervention or control [19]. As such, 10 zones were allocated to the Yathu Yathu intervention. Prior to implementation of the Yathu Yathu intervention, the two study communities were enumerated from September 2019 to January 2020. The initial trial protocol indicated that the pilot phase was months 1-5, after which an adaptation was planned, and full implementation of the intervention would be measured from months 6 – 17 resulting in 12 months of full implementation.

### Yathu Yathu

Yathu Yathu consists of two key intervention components: (i) spaces (hubs) located within the community away from the government-run health facility in each of the ten intervention zones and (ii) a prevention points card (PPC). Through the Yathu Yathu hubs, SRH services are provided by male and female peer support workers (PSWs), a supervisor, who is an experienced community health worker, and a nurse, who provides services once a week at each of the ten hubs. Services provided include: HTS (finger-prick-Determine® or HIV self-testing [HIVST]-Oraquick®), STI screening (with referral to the local government-run health facility if symptomatic), condom distribution, and information and provision of contraceptives. Information and education on SRH is provided through Edutainment (e.g. MTVShuga©) and CSE sessions. In October 2019, social media platforms, including Facebook©, via an open Yathu Yathu Facebook page, and hub-specific WhatsApp© groups, were launched to provide staff a communication route with participants on study-related announcements.

The PPC incentivizes service use by allowing AYP to gain points for services accessed, which can then be redeemed for rewards (primarily health-related rewards, e.g. toothbrush, toothpaste, soap or exercise books) [19]. The PPC additionally allows for tracking frequency and type of services accessed (including: multiple visits, type and number of services accessed per visit and characteristics of AYP accessing services). During enumeration, AYP in intervention zones were informed that they could access and choose any SRH services and accrue points for services accessed at the hubs or the local government-run health facility.

Additionally, the intervention includes regular community engagement about the study through door-to-door mobilization and monthly community meetings with AYP, community leaders such as church leaders and parents/guardians.

### COVID-19 and Yathu Yathu

**Fig. 1:**
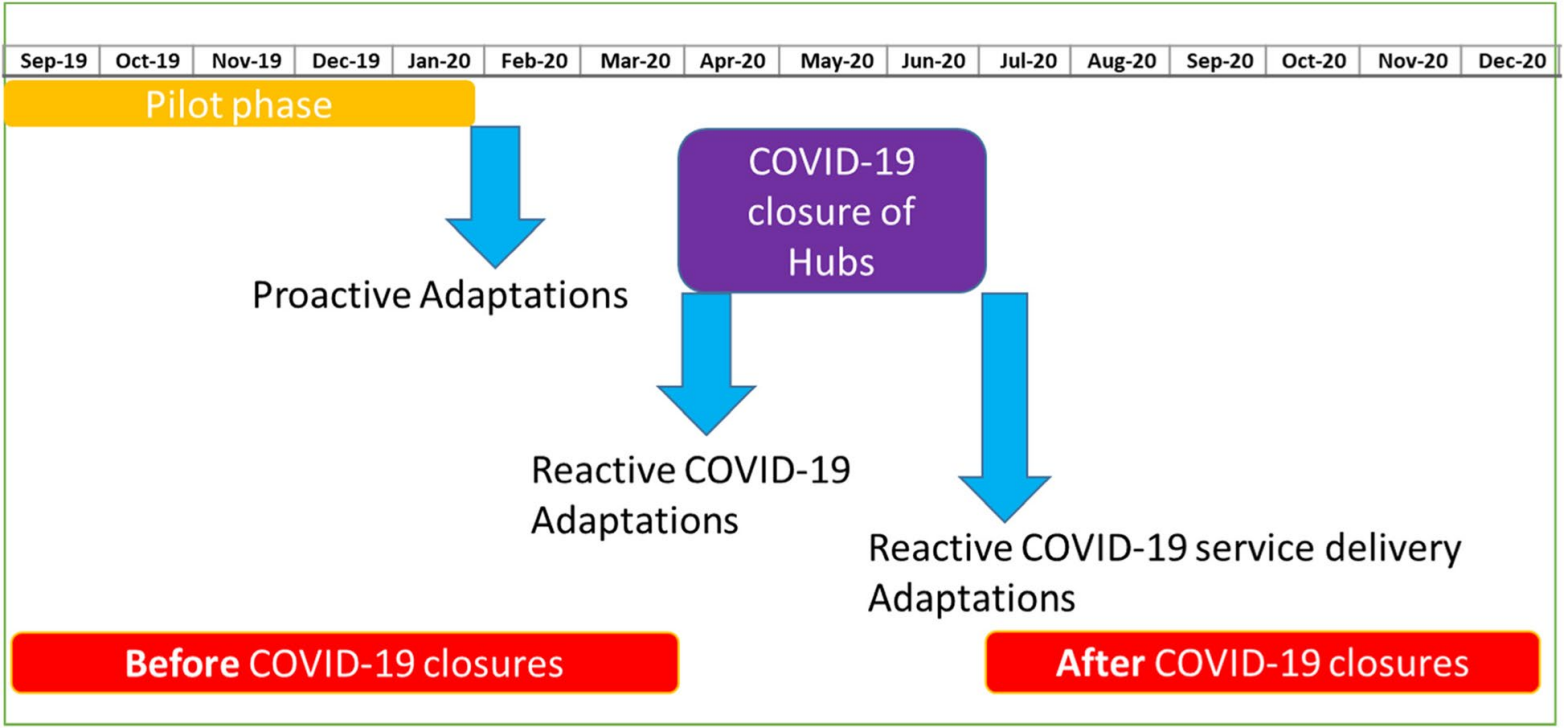
Diagrammatic description of the timings of the COVID-19 related adaptations and the data collection periods

Before Zambia reported any COVID-19 cases, a study contingency plan was developed to guide actions based on outbreak progression, to be followed in the absence of guidance from the Zambia Ministry of Health (MoH) and/or Zambia National Public Health Institute (ZNPHI). After the first case of COVID-19 was reported, and subsequent monitoring of cases, study leadership decided to close all ten hubs and stop all service delivery, including HTS. Hubs were closed 1^st^ April 2020. This triggered the first reactive adaptation to Yathu Yathu. Service delivery was resumed on 1^st^ July 2020 and this resulted in the second reactive adaptation [20] (Fig 1).

### Data collection

For this analysis, we used data collected from September 2019-December 2020, including the pilot phase. COVID-19 closures were from April-July 2020, providing a period “before COVID-19 closures” from September 2019-March 2020 and a period “after COVID-19 closure” from July 2020- December 2020. We report only on reactive adaptations.

Quantitative data was collected routinely via the PPC and Facebook page. Facebook data included the number of followers and the number of interactions with posts from April to December 2020. Qualitative data was collected using observations of service delivery (n=10); interviews with young people accessing services at the hubs (n=4) and focus group discussions (FGDs; n=10), to assess experiences and perceptions of service delivery. This data was collected after the COVID-19 closure. WhatsApp reports on number of participants, topics discussed and questions raised were submitted per community between April 2020- December 2020.

### Data analysis

For this analysis, we used PPC data from AYP residing in the intervention zones only. Using this data, we described the total number of visits to hubs and average number of visits per month for key HIV prevention services: CSE, Edutainment, HTS and STI screening services, before (7 months) and after the COVID-19 closures (6 months).

For both time periods, we described the number of AYP accessing hubs for the first time and uptake of HTS at first visit overall, by age and sex. For uptake of HTS, we exclude those self-reporting they were HIV-positive, as they were not eligible for HIV testing. Subsequently, we describe uptake of HTS by type of HIV test used, which is finger-prick HIV testing or HIVST. Using population-averaged logistic regression to account for clustering by zone, we estimated whether there was evidence for a difference in uptake of HTS at first visit before and after closures overall, for male and female AYP disaggregated by age, separately. We used the period before closures as the reference period and obtained an odds ratio (OR) from the population-averaged logistic regression model.

Facebook data analysis included a review of available Facebook data analytics. We summarized the total number of posts made and uploaded onto the Yathu Yathu page. For each post, we described the “reach” of the post, defined as the number of people who saw the post at least once, and “engagement” with the posts, defined as the number of people that liked, commented, shared or clicked on the post.

Qualitative data was analysed using thematic analysis, specifically the analysis utilized the Rigorous and accelerated Data Reduction technique [21]. WhatsApp group reports containing the number of participants in the groups disaggregated by age and sex, and including the list of topics discussed as well as challenges encountered were reviewed and analysed using a narrative synthesis approach.

## Results

### Description of service delivery adaptations to COVID-19

During hub closures, the *first reactive adaptation* [20] was made. This involved provision of CSE sessions via Facebook and WhatsApp. The nurses and PSWs video recorded sessions and uploaded these onto the social media platforms. COVID-19 information videos were promoted, including ‘how to make a mask’ and an interview with a young person who had COVID-19. Overall, there were 316 Facebook posts from September 2019-December 2020. Of these, 105 (33.2%) were CSE and 6 (1.9%) were COVID-19 videos. The number of Facebook page followers increased from 539 in April 2020 to 891 in June 2020, with the highest increase in new followers in April (n=222) when hubs first closed. By December 2020, the number of followers was 1,174.

The SRH videos with the highest reach included videos on: contraceptives (reach n=2,400, engagement 7.0% n=170/2400), effects of teenage pregnancy (reach n=2,400, engagement 9.5% n=227/2400) and voluntary medical male circumcision (reach n=2,400, engagement 5.8% n=138/2400). The COVID-19 videos had high reach, with the ‘how to make a mask’ video reaching n=122,800 (engagement n=520; 0.4%) and the interview a reach of n=121,400 (engagement n=334; 0.3%).

Before closures, there were 44 participants in WhatsApp groups. By December 2020, there were 435 participants. Of these, 52.6% (n=229/435) were aged 20-24 and 45.1% (196/435) were male and 55.2% (240/435) were female. The five most common topics discussed were: STIs, Post Exposure Prophylaxis, Pre-Exposure Prophylaxis, contraceptives and COVID-19. Reported challenges to participation were that adolescents aged 15-19 were less able to purchase data bundles to access the internet and less likely to have smart phones than young people aged 20-24.

When hubs were opened, a *second round of reactive adaptation*s were implemented to reduce the risk of COVID-19 transmission among study staff and participants. Adaptations included: *(i)* infection control standard operating procedures to guide service delivery, *(ii)* staff provided with personal protective equipment, *(iii)* all hubs required to have a “reception” desk outside where participants were provided information about COVID-19, including transmission and symptoms, temperature checks, and asked if they had any COVID-19 symptoms as guided by MoH/ZNPHI [22]. Any participant with a symptom was supported to call the national toll free line for assistance and not allowed in the hub, *(iv)* contact tracing register was completed and *(v)* a fabric mask provided to all participants before entry. Additionally, *(vi)* hand washing stations were placed at the reception desk, with all participants required to hand wash before entry. *(vii)* The number of participants allowed into hubs was reduced to a maximum of 6 participants, dependent on the size of the hub, to ensure a minimum of 1.5 meters between all individuals, *(viii)* CSE and edutainment sessions were cancelled, as these sessions were popular thus posing a risk to participants and staff, and *(ix)* an appointment system was established. Adaptations did not affect individually accessed services, including HTS. Community mobilization was scaled down and community meetings followed the same requirements as the hubs.

All staff were trained on the adaptations before implementation. Simultaneously, planned consultative meetings were held with participants and adolescent community advisory board members on the adaptations. During these meetings, young people and community members initially expressed their support for the cessation of CSE and edutainment sessions at the hub. However, after a month of implementation, they requested for resumption of these sessions to encourage hub attendance.

### Comparison of uptake of SRH services, including HTS, before and after COVID-19 adaptations

Between September 2019 - December 2020, AYP made 45,968 hub visits. These visits included participants visiting more than once. There were an average of 4,126 visits/month before hub closures, this reduced to 2,848 visits/month after closures and in response to restrictions on the number of AYP able to attend the hubs at any one time. Over the study period, adolescents aged 15-17 made more visits per month than other age groups (Table 1).

**Table 1:** Average number of visits^a^ per month made by adolescents and young people aged 15-24 years old to access specific services in Yathu Yathu hubs before (Sep 2019-March 2020) and after (July 2020-Dec 2020) COVID-19 closures in two urban communities in Lusaka, Zambia.(*N*=46, 760)

Before COVID-19 closures (services accessed average/month)						After COVID-19 closures (services accessed average/month)				
	Overall (28,880 visits)	CSE	Edutainment	HIV testing	STI screening	Overall (17,880 visits)	CSE	Edutainment	HIV testing	STI screening
Overall access										
	**4,126**	**2,414**	**797**	**716**	**181**	**2, 848**	**950**	**272**	**963**	**813**
Adolescent girls and young women										
Overall	**2,986**	**1,525**	**527**	**457**	**113**	**1,995**	**651**	**191**	**647**	**532**
15-17	**1,316**	803	265	214	46	**931**	320	98	272	190
18-19	**657**	384	125	106	29	**442**	143	42	224	124
20-24	**713**	337	136	136	37	**621**	189	51	224	219
Adolescent boys and young men										
Overall	**1440**	**889**	**270**	**259**	**69**	**854**	**299**	**80**	**323**	**280**
15-17	**723**	454	141	126	28	**431**	158	44	144	114
18-19	**279**	238	69	66	19	**206**	73/	17	84	58
20-24	**338**	198	60	67	22	**216**	69	20	94	94

^a^includes multiple visits by a single individual.

Before hub closures, CSE was the most frequently accessed service (2,414 visits/month) followed by Edutainment (797 visits/month). After closures, HIV testing was the most accessed service (963 visits/month), followed by CSE (950 visits/month). Visits for STI screening increased from 181 visits/month to 813 visits/month. Before hub closures, male and female AYP aged 15-19 (male-1,002 visits/month; female-1,973 visits/month) had more visits/month compared to those aged 20-24(male-338 visits/month; female 713 visits/month). After hub closures, AYP aged 15-19 still had more visits/month compared to those aged 20-24(Table 1).

Over the entire study period, 9,436 individuals accessed hubs for the first time. Excluding individuals who self-reported knowing their HIV positive status (150/9,547; 1.6%); 65.3% (n=6,064/9,286) AYP accessed hubs before closures and 34.7% (n=3,222/9286) accessed hubs after closures. Before closures, on average, 866 AYP accessed hubs each month, compared to 581/month after closures.

Before hub closures, overall uptake of HTS was 65.9% (3,993/6,064) compared to 73.2% (2,359/3,222) after closures (Table 2). There was weak evidence for higher acceptance of HTS at first visit after closures (adjOR=1.24, 95%CI 0.99, 1.56, p=0.06). There was evidence that uptake of HTS was higher among young men aged 18-19 after closures relative to before (77.5% vs. 68.3%; adjOR=1.35, 95%CI 1.11, 1.64; p=0.002). Before closures, among all AYP testing for HIV, 78.3% (3,125/3,993) tested using the finger-prick method and 22.1% (882/3993) used HIVST at the hubs. After closures, 91.0% (2,243/2,464) tested using finger-prick and 5.2% (129/2,464) used HIVST at the hubs.

**Table 2:** Average number of AYP accessing Yathu Yathu hubs per month and % choosing to test for HIV at their first visit before (Sep 2019-March 2020) and after (July 2020-Dec 2020) COVID-19 closures in two urban communities in Lusaka, Zambia

Access hubs for the first time and uptake of HIV testing at the first visit of ALL participants aged 15-24 before and after the COVID -19 hub closure (*N*=9,286^a^)						
Overall	**Number/month accessing hubs before closure**	**Number/month accessing hubs after closure**	**Number (%) HIV testing before closure**	**Number (%) HIV testing after closure**	**Odds Ratio**^b^ **(95% CI)**	***p*-value**
	866/month	581/month	3,993/6,064 (65.9%)	2,359/3,222 (73.2%)	1.24 (0.99,1.56)	0.06
Access hubs for the first time and uptake of HIV testing at the first visit of adolescent girls and young women aged 15-24 before and after the COVID -19 hub closure (*N*=5, 932)						
	**Number/per month accessing hubs before closure**	**Number/per month accessing hubs after closure**	**Number (%) HIV testing before closure**	**Number (%) HIV testing after closure**		
Overall	549/month	349/month	2,518/3,841 (65.6%)	1,524/2,091 (72.9%)	1.27 (0.94, 1.73)	0.13
15-17	247/ month	97/month	1,150 /1,728 (66.5%)	439/583 (75.3%)	1.34 (0.94, 1.90)	0.10
18-19	126/ month	77/month	585 / 884 (66.2%)	349/461 (75.7%)	1.36 (0.86, 2.13)	0.19
20-24	176/month	175/month	783/1229 (63.7%)	736/ 1,047 (70.3%)	1.21 (0.89, 1.65)	0.23
Access hubs and for the first time and uptake of HIV testing at the first visit of adolescent boys and young men aged 15-24 before and after the COVID -19 closure (*N*=3,354)						
	**Number/month accessing hubs before closure**	**Number/month accessing hubs after closure**	**Number (%) HIV testing before closure**	**Number (%) HIV testing after closure**		
Overall	318/month	189/month	1,475/2,223(66.4%)	835/1,131 (73.8%)	1.20 (0.97, 1.49)	0.10
15-17	156/ month	58/month	715/1,095 (65.3%)	251/349 (72.0%)	1.23 (0.95, 1.58)	0.11
18-19	79/ month	50/month	377/552 (68.3%)	234/302 (77.5%)	1.35 (1.11, 1.64)	0.002
20-24	82/ month	80/ month	383/576 (66.5%)	350/480 (73.0%)	1.13 (0.80, 1.59)	0.50

^a^ excluding those that self-reported HIV positive at first visit

^b^ Adjusted for clustering by zone

### Experiences of accessing services after COVID-19 adaptations

Observations and FGDs revealed that AYP were disappointed with restrictions on the numbers of participants allowed to enter the hub, as this meant they could not attend with their friends.

*Unless when corona ends that is when we can come in a group, a long time ago we used to enjoy because we would be many, someone this side gives their opinion and another on the other side gives a different opinion … but now we are few so even that services is not very entertaining. (Young man, FGD 20 – 24) Z9*

However, some young people, particularly, those older than 20, welcomed and preferred small groups for CSE and edutainment sessions.

*I think it is just fine because, where there are few people, it helps one to understand compared to where there are many people. You find that when you are many, your friends might be talking loudly but if you are few it will even be easier … you ask questions and the interaction is easier (young man, 15 – 19 FGD) Z8.*

In addition, social media-related adaptations for CSE, especially the use of WhatsApp, were welcomed by those aged 20-24:

*Yes the whatsapp group is helpful, it is very helpful because you find that you were busy with something then when you are only on whatsapp you find a particular topic, your friends have been discussing about the same topic … you are free to contribute or to ask any question on that same topic. It does not have a limited time that discussion on this topic is over, there is nothing like that, even if you go online at 22:00 hours, you ask, they will even answer you in the morning (young woman, FGD 20 – 24) – Z9.*

Despite some disappointments, interviewees expressed happiness that the hubs reopened

*” … . [we are] still appreciative of services and learning about health including COVID 19 … .” (young man, IDI, 21, Z9),* with other participants expressing understanding for the restrictions to the hub “ … *I think what they are doing they are trying to protect us because of the Covid, it’s not like they just want to restrict us or what, they are trying also to look after our health and also their health, that’s why … ” (Young woman, FGD 20 - 24 years, Z8).*

Observations revealed that the appointment system did not work well; some participants made appointments but did not attend, resulting in others having to wait as sessions were already “filled”. Additionally, although masks were provided on a one-off basis, AYP often arrived without masks, citing various reasons for not having their mask, including having lost the mask, lent the mask to a relative, or just not wanting to wear a mask.

## Discussion

Despite adaptations in response to COVID-19 that resulted in restrictions in attendance, in the period after COVID-19 related closures, over 3,000 AYP visited the Yathu Yathu hubs for the first time. Approximately 73% accessed HTS, and attendance to social media platforms increased during the COVID-19 closure. Even with restrictions due to COVID-19, HIV testing was the most accessed service and uptake of HTS at first visit remained high. These findings show that HTS can be provided during COVID-19 without affecting demand. Qualitative data showed that, while AYP were disappointed by the restrictions, they understood the need for the restrictions and were happy with continued service provision, including via social media.

Adaptation, defined as a “process of making modifications or changes to the design or implementation of an intervention”, is important for optimizing implementation [10, 11]. Adaptations are important for meeting a specific target group needs or as a response to a contextual stimulant, such as disease outbreaks [12]. However, adaptations can result in changes to the fidelity of the intervention, reach and the dose delivered and received [13]. This is evidenced in Yathu Yathu, with AYP not accessing services due to adaptations made to limit the number of participants and so impacting reach and dose received among those reached. On the other hand, adaptations may be necessary to maintain the integrity of an intervention due to unforeseen circumstances, such as COVID-19. In the case of Yathu Yathu, with the simple service delivery adaptations described, we have shown that community-based provision of SRH services is safe, feasible and acceptable to AYP even in the midst of an epidemic. Another adaptation was use of social media platforms, which have already been documented as a useful way to share SRH information in Zambia, Kenya, South Africa and South Asia [23–25]. In Yathu Yathu, social media evolved to a platform to provide CSE in response to restrictions on in-person provision of this service. With a changing social context and more young people (71%) reported to be on social media compared to those aged over 24 (48%) [24], this presented an important opportunity to share information. However, in Sub-Saharan Africa, use of mobile phones is still limited, with only 20% of the population owning a phone compared to Europe and North America at 78% [24]. Concerns about creating a “digital divide” due to lack of access to smartphones, was evidenced by the lower number of adolescents aged 15-17 engaging with the virtual platforms. This, thus, highlights the importance of continued physical access to ensure that this key age group is not left behind in their access to critical SRH services. Here, we also see that young people aged 20-24, who are more likely to be economically independent, and so have greater access to social media, were able to benefit more. This is similar to findings from countries such as South Africa, Nigeria and Ghana as part of the SmartSex social media platform for provision of sexuality education [25]. On the other hand, this age group had fewer visits to the hubs than the adolescents aged 15-17, with some citing “being busy” and therefore found this avenue as a “helpful” addition to hub services.

Although adaptations made in response to COVID-19 resulted in reduced numbers of participants visiting the hubs and potentially without their friends, AYP still welcomed the availability of the services and were not discouraged from accessing services. Before the COVID-19 closures, adolescent boys and girls aged 15-19 accessed more services compared to those aged 20-24 years. This was seen even after closures. Considering that, in Zambia, this age group has the lowest correct knowledge of HIV (˜40%) and between 45%-60% report never having tested for HIV [6], having access to SRH services, may be key in ensuring gains made in HIV infection reduction targets are maintained [1, 26, 27]. Encouragingly, for this underserved age group, more girls than boys consistently accessed services. This was also seen for the young women aged 20-24 years compared to young men. Despite young men not accessing services as often as women, the proportion accessing HTS was similar across both sexes implying that the hubs are acceptable for both to access services. This is in contrast to other studies that found that attendance to spaces was dominated by young men [7, 28]. Surprisingly, HIVST uptake did not increase as expected after COVID-19 related closures [29]. This could be explained by shortages in HIVST test kits, which were sourced from government health facilities, during implementation. In addition, this could be explained by findings from previous analysis of our pilot intervention data, which suggests that provision of youth friendly services results in a preference for fingerprick HIV testing [30]. The increased number of visits for STI screening and HTS may be distorted by the PPC component of the intervention, where AYP may wish to “gain” points to be able to access rewards as opposed to a perceived need for the service itself. On the other hand, the increase may be due to reduced access to services, such as condoms, during closures, and/or increases in sexual violence, coupled with closure of schools, resulting in increased exposure to HIV and STIs [16, 31].

Our study is subject to limitations. It was not possible to determine if the individuals reached through the Facebook page were study participants who received a PPC, as there were no restrictions on who could engage with the page. As such, the increase in followers may not reflect an increase in the reach among our study population. As COVID-19 resulted in a number of restrictions including to information on sexual and reproductive health, we felt it would be unethical to not provide information to all AYP. This would have resulted in a higher number of new followers who were not necessarily from our study area thereby distorting the usefulness of social media to our study. This could be overcome by having a restricted group only open to participants if needed. However, most followers were young people in our target age group, residing in Lusaka. Another limitation is that, as we did not compare uptake of HTS in the hubs to uptake of HTS in the control zones (health facilities). As such, we do not know whether AYP would have had access to HTS elsewhere and cannot attribute the increase in uptake of HIV testing at population level to the COVID-19 closures. COVID-19 control measures may have resulted in limited access to healthcare services, including HTS, at local health facilities. This could have positively influenced access to HTS at the hubs once they re-opened. However, we cannot attribute the increase in uptake of HTS to the re-opening of hubs as we cannot determine what could have happened in the absence of closures. To overcome this limitation, we restricted the analysis to uptake of HTS at the very first visit to the hubs to ensure we didn’t include AYP already engaged with the hubs; particularly as other research has shown that uptake of HTS is associated with more frequent use of health services in young people aged 15-24yrs old in a similar setting [32]. However, this analysis is planned at the end of the trial, which was extended by an additional 10 months due to COVID-19. The strengths of this research are that we collected and used real-time data on adaptations in a complex and changing environment. The PPC is a valuable resource which allowed us to collect this data.

## Conclusion

As the world battles COVID-19, ensuring AYP have access to essential SRH services and information has become more crucial to maintain the gains made in adolescent health in Sub-Saharan Africa [33, 34]. Making simple adaptations, such as enforcing social distancing and infection control protocols, has been shown to work in maintaining access to SRH in countries like Uganda [23]. With continued visits and uptake of SRH services, we have shown that making simple adaptations to service delivery of SRH services within a community-based setting, with additional use of social media to support information provision, can successfully ensure continued provision and use of SRH services and HTS by AYP.

## Data Availability

The datasets generated and/or analysed during the current study are not publicly available due to participant confidentiality but are available from the corresponding author on reasonable request.
